# Use of lymphoscintigraphy to differentiate primary versus secondary lower extremity lymphedema after surgical lymphadenectomy: a retrospective analysis

**DOI:** 10.1186/s12957-018-1379-5

**Published:** 2018-04-10

**Authors:** Mirela Mariana Roman, Romain Barbieux, Jean-Marie Nogaret, Pierre Bourgeois

**Affiliations:** 1 Department of Mammo-Pelvic Surgery, Jules Bordet Institute, 121, Bd. de Waterloo, 1000 Brussels, Belgium; 2 Service of Kinesitherapy, Jules Bordet Institute, Université Libre de Bruxelles, Brussels, Belgium; 3 Service of Nuclear Medicine, Jules Bordet Institute, Université Libre de Bruxelles, Brussels, Belgium; 4 Multidisciplinary Clinic of Lymphology, Jules Bordet Institute, Université Libre de Bruxelles, Brussels, Belgium

**Keywords:** Cancer-related lymphedema, Lower limb, Lymphoscintigraphy, Primary lymphedema, Secondary lymphedema

## Abstract

**Background:**

When managing patients with cancer, lymphedema of the lower limbs (LLL) is commonly reported as secondary to the surgical excision and/or irradiation of lymph nodes (LNs). In the framework of lymphoscintigraphic imaging performed to evaluate secondary LLL, some lympho-nodal presentations have been observed that could not be explained by the applied treatments, suggesting that these LLL might be primary. Therefore, all our lymphoscintigraphic examinations that were performed in patients for LLL after surgery for gynecological or urological cancer were retrospectively analyzed in order to evaluate the frequency in which these LLL might not be secondary (either completely or partially) but primary in origin.

**Methods:**

Lymphoscintigraphies performed in 33 patients who underwent LN dissection (limited to the intra-abdominal LN) with or without radiotherapy for histologically confirmed ovarian cancer (*n* = 6), uterine cancer (*n* = 14 with cervical cancer and *n* = 7 with endometrial cancer), or prostate cancer (*n* = 6) were compared to lymphoscintigraphies obtained in primary LLL.

**Results:**

In 12 (33% of the) patients (3 men plus 9 women, 4 with cervical cancer and 5 with endometrial cancer), scintigraphy of the lower limbs revealed lympho-nodal presentation that did not match with the expected consequences of the surgical and/or radiological treatments and were either suggestive or typical of primary lymphedema.

**Conclusions:**

This retrospective analysis of a limited but well-defined series of patients suggests that the appearance of LLL might not be related to cancer treatment(s) but that these LLL may represent the development of a primary lymphatic disease latent prior to the therapeutic interventions.

## Background

For many patients with gynecological cancer and urological cancer, lower limb lymphedema (LLL) is one of the most disabling secondary effects of the surgical and/or radiotherapeutic treatment [1–3]. In this context, LLL is related to lymph node (LN) dissection (iliac, obturator fossa, presacral nodes for the prostate cancer and iliac, obturator, sacral, pararectal, paraaortic for gynecologic malignancy) [4, 5]. However, LLL can also be primary. When primary lymphedema is investigated using scintigraphy (i.e., by lymphoscintigraphy), the imaging may sometimes mimic what is observed in secondary cases (e.g., after inguinal lymphadenectomy for melanoma). On the other hand, the authors struggled with the fact that some lymphedemas investigated after such limited lymphadenectomies for cancer did not have imaging typical of the consequences of these surgeries (i.e., the absence of visualization of lymph nodes in the operated areas and with their consequences at the level of the lower limbs) but one imaging rather in agreement with what can be observed in primary lymphedemas (with normal visualization of the intra-abdominal lymph nodes but with abnormalities at the level of the inguinal lymph nodes, at the root of the lower limbs and/or at the level of the distal part of the limbs). The aim of the present paper is to report a retrospective analysis of well selected patients in which LLL appeared after limited lymph node dissection (iliac, obturator, paraaortic, sacral, pararectal) in order to evaluate the frequency in which these LLL might not be, either completely or partially, secondary to the consequence of surgery, but primary in origin.

## Methods

### Patients

Twenty-seven women (14 with cervical or uterine cancer, 6 with ovarian cancer, and 7 with endometrial cancer; mean age 58 years, range 33 to 84 years) and 6 men (with carcinoma of the prostate; mean age 71 years, range 68 to 77 years) who had undergone lymphoscintigraphic investigation of secondary LLL at the Department of Nuclear Medicine in our hospital from August 2009 to July 2016 were retrospectively analyzed. The LLL was unilateral in 25 patients and bilateral in 8 patients. The delay between surgery and the lymphoscintigraphic investigation ranged from 3 to 312 months. Patients with evolutive cancerous disease were excluded.

### Lymphoscintigraphic technique

Radionuclide lower limb lymphangiography was performed according to a well-standardized protocol [6]. One tenth of one vial of human serum albumin nanosized colloids (Nanocoll R, GE Healthcare, Belgium) labeled with 2 mCi (74 MBq) of ^99m^Tc in a volume of 0.2 ml was injected subcutaneously into the first interdigital space of each foot. Using a dual-head single-photon gamma camera equipped with a parallel-hole all-purpose low-energy collimator, planar whole body scanning (WBS) images (anterior and posterior views) were obtained from the feet to the head after a succession of three phases: after 30 min the limbs in resting position (phase 1), after 5 min of tip-toeing (phase 2), and after 1 h of normal activity (phase 3). Dynamic images centered on the inguinal area during phases 1 and 2 as well as one SPECT-CT after phase 3 were also performed as parts of the protocol. In patients for whose the tracer had not reached (and did not show) the inguinal LN within the limits of these three phases (after phase 3), one additional injection (0.4 ml from the same vial as for the injections in the feet) was also performed intradermally in the lateral part of the buttock in front of the great trochanter in order to force the visualization of the inguinal and/or intra-abdominal LN (phase 4 of our protocol). In the present study, only the pictures obtained after phase 3 (and phase 4 if performed) were analyzed (with demonstration or not of the LN and of all the other abnormalities related to lymphedema).

## Results

Among the 33 patients with LLL, 12 (33%; 3 men and 9 women, 4 with cervical cancer and 5 with endometrial cancer: see Table 1) had lymphoscintigraphic results for the lower limbs that did not match the expected consequences of the limited intra-abdominal lymphadenectomy.

**Table 1:** Patient characteristics

*N*	Cancer type	LNs dissected	LNs metastatic	RT	LLL
1	Prostate	24 (14R/10L)	1	Yes	R
2	Prostate	12 (8R/4L)	0	Yes	L
3	Prostate	16 (9R/7L)	0	Yes	R–L
4	Endometrial	41 (10R/10L/21PA)	0	No	L
5	Endometrial	8 (4R/4L)	1	No	R–L
6	Endometrial	8 (ND)	0	No	R
7	Endometrial	24 (16R/8L)	0	No	R
8	Endometrial	12 (4R/8L)	0	No	R–L
9	Cervical	28 (ND)	0	Yes	R
10	Cervical	12 (ND)	0	Yes	R
11	Cervical	20 (8R/12L)	0	No	L
12	Cervical	ND	ND	Yes	L

*ND* no data, *LN* lymph node, *RT* radiotherapy, *R* right, *L* left, *PA*, para-aortic

**Fig. 1:**
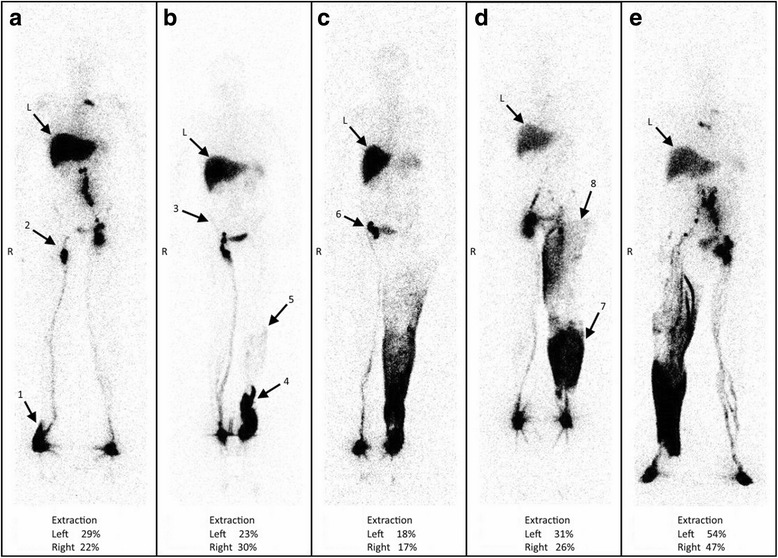
**a**–**e** Anterior whole-body scintigraphy performed at the end of our protocol (after 1 h of normal activities) in five patients with lower limb lymphedema who underwent bilateral lymphadenectomy (iliac and obturator). These images illustrate the 12 patients in whom we had discrepancies between what was observed and the expected consequences of the surgeries regarding lymphatic and nodal abnormalities. Arrows with L indicate the liver, wherein radiocolloids are taken up when they have reached the systemic circulation

Figure 1 shows representative images of such discrepancies obtained in five of these patients. Figure 1a is the image of a woman who had undergone radical hysterectomy with bilateral pelvic lymph node dissection for cervical cancer 6 years before the lymphoscintigraphic examination and developed right LLL 12–18 months after surgery. The lympho-nodal images match the consequences of the surgery on the left side (with common iliac gap) but not on the right side (with progression of the tracer in the dermal collateralization lymphatic network limited to the distal part of the foot (arrow 1) and with only the most inferior inguinal lymph node (arrow 2) and no other right-sided infra-diaphragmatic lymph node). In Fig. 1b, the lymphoscintigraphy was performed 6 months after radical hysterectomy with bilateral pelvic lymph node dissection for cervical cancer and 15 days after the appearance of left LLL. Normal lymphatic drainage is observed at the level of the (nonedematous) right limb with visualization of the inguinal lymph node but no intra-abdominal lymph node (arrow 3), whereas on the left side progression of the tracer thorough the dermal superficial collateralization network is only seen limited to the foot and ankle (arrow 4) and up to under the knee (arrow 5) without visualization of any inguinal and/or iliac lymph nodes. Figure 1c was also obtained in a woman 11 years after radical hysterectomy with bilateral pelvic lymph node dissection for cervical cancer and 9 years after the appearance of left LLL. On the left side, lymphatic drainage is seen through the dermal superficial collateralization network up to the mid-part of the thigh and no lymph nodes are seen. In her case, at the level of the nonedematous limb, normal lymphatic vascular drainage is observed up to the root of the limb, but with only inguino-crural lymph nodes (arrow 6) and without inferior inguinal or intra-abdominal lymph nodes. In Fig. 1d, the lymphoscintigraphy was performed 18 months after radical hysterectomy with bilateral pelvic lymph node dissection for cervical cancer and 10 months after the appearance of left LLL. Normal lymphatic drainage is observed at the level of the right limb with visualization of the inguinal and intra-abdominal lymph nodes, whereas on the left side dermal backflows (the term “dermal backflow” is used when vascular reflux of lymph is seen originating from LN and/or lymph vessel and when the tracer reaches the dermal superficial collateralization network) are seen at the level of the calf (arrow 7) and from the inguinal lymph node into the upper two thirds of the thigh (arrow 8). In Fig. 1e, the examination was performed 5 years after radical prostatectomy with bilateral extended pelvic lymph node dissection and 2 months after the appearance of right LLL. Normal lymphatic drainage is observed at the level of the left limb with visualization of the inguinal and intra-abdominal lymph nodes, whereas on the right side dermal backflows are seen from the foot to the knee with collecting lymphatic vessels at the level of the thigh and through the inguinal area (without intercalated lymph nodes), before reaching the right iliac lymph nodes.

## Discussion

Secondary lymphedema is the most prevalent form of lymphedema and represents a serious complication and chronic disease, lasting a lifetime in most cases [7]. The incidence rate of LLL that originates in lymphadenectomy has been reported to be 7–36% in patients with any gynecological cancer [8–14] but more precisely 1–38% for patients with endometrial cancer [2, 4, 15–17], 17–81% with cervical cancer [18–24], 6–75% with vulvar/vaginal cancer [25–30] and 5–21% with ovarian cancer [8, 9, 13]. After treatment for prostate cancer, pooled lymphedema incidences was 4% but 16% among patients who received radiotherapy [31].

The traditional standard- for imaging the lymphatic system is lymphoscintigraphy. Lymphoscintigraphy is a reliable, objective, and noninvasive means of supporting the diagnosis of lymphedema [32].

In the present limited series, the lymphoscintigraphic images did not show in quite one third of the patients the consequences of the iliac and of the obturator lymphadenectomies performed in these patients. Instead, the absence of inguinal lymph nodes that were not removed and/or the presence of lymphatic reflux from the inguinal lymph nodes were noted and these images represent relatively typical presentations for primary lymphedemas.

**Fig. 2:**
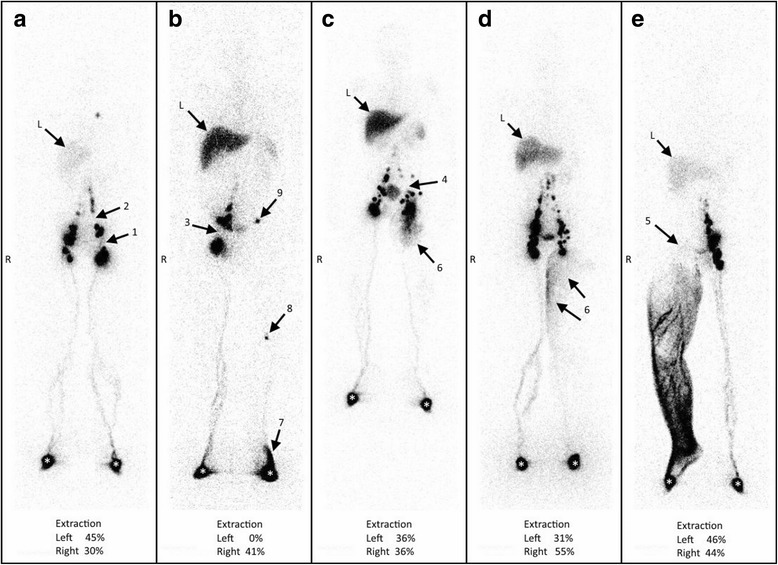
**a**–**e** Anterior whole-body scintigraphy performed at the end of our protocol (after 1 h of normal activities) in five patients with primary lower limb lymphedema. Arrows with L indicate the liver, wherein radiocolloids are taken up when they have reached the systemic circulation

Figure 2 shows some examples of primary lymphedemas. Limited lympho-nodal “gaps” (lymphadenodysplasia) are visible in Fig. 2a at the level of the inguinal (arrow 1) and common iliac (arrow 2) groups on the left side, in Fig. 2b at the level of the right inguinal group (arrow 3), and in Fig. 2c at the level of the left iliac group (arrow 4). Figure 2e shows the consequences of extensive inguino-iliac lymphadenodysplasia (the inguino-iliac lymph nodes are not visible, arrow 5) on the right side with the tracer flowing into lymphatic vessels and the superficial lymphatic collateralization network up to the root of the limb. Vascular lymphatic reflux was also observed from the left inguinal lymph nodes in Fig. 2c, d (arrows 6). The situation at the left lower limb in Fig. 2b can be classified as the consequences of one lymphangiodysplasia with an absence of normal superficial lymphatic vascular drainage and the tracer flowing from the injected interdigital space through the superficial lymphatic collateralization network at the level of the dorsum of the foot (arrow 7) with one popliteal lymph node (arrow 8) intercalated on the deep lymphatic system draining the limb and reaching the inguino-crural lymph node (arrow 9) without visualization of the inguinal lymph nodes. Comparing the two sets of figures, Fig. 1d can be compared to and superimposed on Fig. 2c, d, Fig. 1c to and on Fig. 2e, Fig. 1e to and on Fig. 2e, Fig. 1b to and on Fig. 2b, and Fig. 1a to and on Fig. 2a, respectively.

These results suggest that these LLL after intra-abdominal lymphadenectomymay be due, at least in part, to underlying and pre-existing latent primary lymphatic disease. Primary lymphedema represents a relatively rare disease that may sometimes be diagnosed without clinically obvious symptoms [33] and several cases were reported to occur after lymphadenectomy [34]. According to Papendieck [35], these lymphedematous situations may result from lympho-(nodal-)adeno-dysplasia and/or lymph-(vessel-)angio-dysplasia. Sustaining the hypothesis of such primary LLL after these intra-abdominal lymphadenectomies, it might be underlined that only one fourth (8) of the (33) patients had bilateral lymphedema while all had undergone bilateral pelvic lymphadenectomy what would have to lead theoretically to bilateral lymphedemas. In breast cancer, the hypothesis of one primary origin has been substantiated for secondary upper limb lymphedema (BCRL). Recent studies have also identified polymorphisms in multiple candidate genes that appear to be associated with the development of BCRL [36, 37]. Bains et al. [38] also reported abnormal lower limb lymphoscintigraphy in two thirds of the women who had developed upper limb BCRL and in half of the women who had no BCRL, suggesting an unidentified association between breast cancer and lymphatic dysfunction.

Our study has the limitations to be retrospective and to analyze a relatively small series of (well selected) patients. Nevertheless, our observations may have several implications. From a general point of view, they may partly exonerate the surgeons in the development of several types of LLL. On the other hand, our observations would lead surgeons who wish to perform lymphadenectomy to pay more attention to patients who present with signs of edema before surgery and/or a familial history of primary LLL. For the best, these patients would have to be investigated by lymphoscintigraphy before surgery in order to exclude the presence of underlying primary lymphatic disease. Some patients might also thereafter benefit from a sentinel lymph node procedure rather than complete lymph node group dissection. The realization of lymphaticovenous anastomosis as proposed by Boccardo et al. [39] in melanoma might also represent an alternative when facing these situations.

## Conclusions

This retrospective analysis of a limited but well-defined series of patients suggests that the appearance of LLL might not be related to cancer treatment(s) (in one third of the cases) but that these LLL may represent the development of a primary lymphatic disease latent prior to the therapeutic interventions. In addition, these situations may be suspected on the basis of lymphoscintigraphic investigations.

## Abbreviations

BCRL: Breast cancer related lymphedema
LLL: Lower limb lymphedema
LNs: Lymph nodes

## References

[1] Soisson AP, Soper JT, Clarke-Pearson DL, Berchuck A, Montana G, Creasman WT (1990). Adjuvant radiotherapy following radical hysterectomy for patients with stage IB and IIA cervical cancer. Gynecol Oncol.

[2] Todo Y, Yamamoto R, Minobe S, Suzuki Y, Takeshi U, Nakatani M, Aoyagi Y, Ohba Y, Okamoto K, Kato H (2010). Risk factors for postoperative lower-extremity lymphedema in endometrial cancer survivors who had treatment including lymphadenectomy. Gynecol Oncol.

[3] Briganti A, Chun KKH, Salonia A, Suardi N, Gallina A, Da Pozzo LF, Roscigno M, Zanni G, Valiquette L, Rigatti P, Montorsi F, Karakiewicz PI (2006). Complications and other surgical outcomes associated with extended pelvic lymphadenectomy in men with localized prostate cancer. Eur Urol.

[4] Abu-Rustum NR, Alektiar K, Iasonos A, Lev G, Sonoda Y, Aghajanian C, Chi DS, Barakat RR (2006). The incidence of symptomatic lower-extremity lymphedema following treatment of uterine corpus malignancies: a 12-year experience at Memorial Sloan-Kettering Cancer Center. Gynecol Oncol.

[5] Clark T, Parekh DJ, Cookson MJ, Chang SS, Smith ER, Wells N, Smith J (2003). Randomized prospective evaluation of extended versus limited lymph node dissection in patients with clinically localized prostate cancer. J Urol.

[6] PB methodological protocol for the scintigrahic investigations of the superficial lymphatic system in patients with limb edemas. 2017. Available from: https://www.bordet.be/fichiers_web/lymphologie/scintigraphic_investigations_superficial_lymphatic_system_with_limb_edemas_2017.pdf.

[7] Boris M, Weindorf S, Lasinski S (1997). Persistance of lymphedema reduction after noninvasive complex lymphedema therapy. Oncol.

[8] Achouri A, Huchon C, Bats AS, Bensaid C, Nos C, Lecuru F (2013). Complications of lymphadenectomy for gynecologic cancer. Eur J Surg Oncol.

[9] Beesley V, Janda M, Eakin E, Obermair A, Battistutta D (2007). Lymphedema after gynecological cancer treatment: prevalence, correlates, and supportive care needs. Cancer.

[10] Dunberger G, Lindquist H, Waldenstrom AC, Nyberg T, Steineck G, Avall-Lundqvist E (2013). Lower limb lymphedema in gynecological cancer survivors–effect on daily life functioning. Support Care Cancer.

[11] Kondo E, Tabata T, Shiozaki T, Motohashi T, Tanida K, Okugawa T, Ikeda T (2013). Large or persistent lymphocyst increases the risk of lymphedema, lymphangitis, and deep vein thrombosis after retroperitoneal lymphadenectomy for gynecologic malignancy. Arch Gynecol Obstet.

[12] Obermair A, Ginbey P, AJ MC (2003). Feasibility and safety of total laparoscopic radical hysterectomy. J Am Assoc Gynecol Laparosc.

[13] Ryan M, Stainton MC, Slaytor EK, Jaconelli C, Watts S, Mackenzie P (2003). Aetiology and prevalence of lower limb lymphoedema following treatment for gynaecological cancer. Aust N Z J Obstet Gynaecol.

[14] Tada H, Teramukai S, Fukushima M, Sasaki H. Risk factors for lower limb lymphedema after lymph node dissection in patients with ovarian and uterine carcinoma. BMC Cancer. 2009; 10.1186/1471-2407-9-47. 10.1186/1471-2407-9-47 PMC2660366 19193243

[15] Konno Y, Todo Y, Minobe S, Kato H, Okamoto K, Sudo S Takeda M, Watari H, Kaneuchi M, Sakuragi N (2011). A retrospective analysis of postoperative complications with or without para-aortic lymphadenectomy in endometrial cancer. Int J Gynecol Cancer.

[16] Kuoppala T, Tomas E, Heinonen PK (2004). Clinical outcome and complications of laparoscopic surgery compared with traditional surgery in women with endometrial cancer. Arch Gynecol Obstet.

[17] Salani R, Preston MM, Hade EM, Johns J, Fowler JM, Paskett EP, Katz ML (2014). Swelling among women who need education about leg lymphedema: a descriptive study of lymphedema in women undergoing surgery for endometrial cancer. Int J Gynecol Cancer.

[18] Bergmark K, Avall-Lundqvist E, Dickman PW, Henningsohn L, Steineck G (2006). Lymphedema and bladder-emptying difficulties after radical hysterectomy for early cervical cancer and among population controls. Int J Gynecol Cancer.

[19] Halaska MJ, Novackova M, Mala I, Pluta M, Chmel R, Stankusova H, Robova H, Rob L (2010). A prospective study of postoperative lymphedema after surgery for cervical cancer. Int J Gynecol Cancer.

[20] Hosaka M, Watari H, Takeda M, Moriwaki M, Hara Y, Todo Y, Ebina Y, Sakuragi N (2008). Treatment of cervical cancer with adjuvant chemotherapy versus adjuvant radiotherapy after radical hysterectomy and systematic lymphadenectomy. J Obstet Gynaecol Res.

[21] Kim JH, Choi JH, Ki EY, Lee SJ, Yoon JH, Lee KH, Park TC, Park JS, Bae SN, Hur SY (2012). Incidence and risk factors of lower-extremity lymphedema after radical surgery with or without adjuvant radiotherapy in patients with FIGO stage I to stage IIA cervical cancer. Int J Gynecol Cancer.

[22] Ohara K, Tsunoda H, Satoh T, Oki A, Sugahara S, Yoshikawa H (2004). Use of the small pelvic field instead of the classic whole pelvic field in postoperative radiotherapy for cervical cancer: reduction of adverse events. Int J Radiat Oncol Biol Phys.

[23] Ohba Y, Todo Y, Kobayashi N, Kaneuchi M, Watari H, Takeda M, Sudo S, Kudo M, Kato H, Sakuragi N (2011). Risk factors for lower-limb lymphedema after surgery for cervical cancer. Int J Clin Oncol.

[24] Hoogendam JP, Verheijen RH, Wegner I, Zweemer RP (2014). Oncological outcome and long-term complications in robot-assisted radical surgery for early stage cervical cancer: an observational cohort study. BJOG.

[25] Carlson JW, Kauderer J, Walker JL, Gold MA, O'Malley D, Tuller E, Clarke-Pearson DL (2008). A randomized phase III trial of VH fibrin sealant to reduce lymphedema after inguinal lymph node dissection: a gynecologic oncology group study. Gynecol Oncol.

[26] Gaarenstroom KN, Kenter GG, Trimbos JB, Agous I, Amant F, Peters AA, Vergote I (2003). Postoperative complications after vulvectomy and inguinofemoral lymphadenectomy using separate groin incisions. Int J Gynecol Cancer.

[27] Hinten F, Van den Einden LC, Hendriks JC, Van der Zee AG, Bulten J, Massuger LF, Van de Nieuwenhof HP, De Hullu JA (2011). Risk factors for short- and long-term complications after groin surgery in vulvar cancer. Br J Cancer.

[28] Novackova M, Halaska MJ, Robova H, Mala I, Pluta M, Chmel R, Rob L (2012). A prospective study in detection of lower-limb lymphedema and evaluation of quality of life after vulvar cancer surgery. Int J Gynecol Cancer.

[29] Sawan S, Mugnai R, Lopes Ade B, Hughes A, Edmondson RJ (2009). Lower-limb lymphedema and vulval cancer: feasibility of prophylactic compression garments and validation of leg volume measurement. Int J Gynecol Cancer.

[30] Walker KF, Day H, Abu J, Nunns D, Williamson K, Duncan T (2011). Do surgical techniques used in groin lymphadenectomy for vulval cancer affect morbidity rates?. Int J Gynecol Cancer.

[31] Cormier JN, Askew RL, Mungovan KS, Xing Y, Ross MI, Armer JM (2010). Lymphedema beyond breast cancer: a systematic review and meta-analysis of cancer-related secondary lymphedema. Cancer.

[32] Szuba A, Shin WS, Strauss HW, Rockson S (2003). The third circulation: radionuclide lymphoscintigraphy in the evaluation of lymphedema. J Nucl Med.

[33] Lee BB, Andrade M, Antignani PL, Boccardo F, Bunke N, Campisi C, Damstra R, Flour M, Forner-Cordero I, Gloviczki P, Laredo J, Partsch H, Piller N, Michelini S, Mortimer P, Rabe E, Rockson S, Scuderi A, Szolnoky G, Villavicencio JL (2013). Diagnosis and treatment of primary lymphedema. Consensus document of the international union of phlebology. Int Angiol.

[34] Bourgeois P, Dargent JL, Larsimont D, Munck D, Sales F, Boels M, De Valck C (2009). Lymphoscintigraphy in angiomyomatous hamartomas and primary lower limb lymphedema. Clin Nucl Med.

[35] Papendieck CM (1999). Lymphatic dysplasia in paediatrics. A new classification. Int Angiol.

[36] Miaskowski C, Dodd M, Paul SM, West C, Hamolsky D, Abrams G, Cooper BA, Elboim C, Neuhaus J, Schmidt BL, Smoot B, Aouizerat BE (2013). Lymphatic and angiogenic candidate genes predict the development of secondary lymphedema following breast cancer surgery. PLoS One.

[37] Newman B, Lose F, Kedda MA, Francois M, Ferguson K, Janda M, Yates P, Spurdle AB, Hayes SC (2012). Possible genetic predisposition to lymphedema after breast cancer. Lymphat Res Biol.

[38] Bains SK, Peters AM, Zammit C, Ryan N, Ballinger J, Glass DM, Allen S, Stanton AW, Mortimer PS, Purushotham AD (2015). Global abnormalities in lymphatic function following systemic therapy in patients with breast cancer. Br J Surg.

[39] Boccardo F, De Cian F, Campisi CC, Molinari L, Spinaci S, Dessalvi S, Talamo G, Campisi C, Villa G, Bellini C, Parodi A, Santi PL, Campisi C (2013). Surgical prevention and treatment of lymphedema after lymph node dissection in patients with cutaneous melanoma. Lymphology.

